# Perception about human papillomavirus vaccination among middle adolescent school girls in Addis Ababa, Ethiopia 2023: qualitative study

**DOI:** 10.1186/s12905-023-02660-1

**Published:** 2023-09-30

**Authors:** Abel Wubu, Bargude Balta, Amsale Cherie, Ketema Bizuwork

**Affiliations:** 1 Addis Ababa University College of health science, Addis Ababa, Ethiopia; 2 Hawassa University College of Medicine and Health Sciences, Hawassa, Ethiopia

**Keywords:** Human papilloma virus, Perceptions, School Girls, Ethiopia

## Abstract

**Background:**

Human papillomavirus vaccine uptake among school girls in Ethiopia was still low and uptake was majorly related to perception regarding the vaccine. This study explored school girls’ perceptions of Human papillomavirus vaccination in Addis Ababa, Ethiopia.

**Objectives:**

The main aim of this study was to explore the perception of secondary school girls towards Human Papilloma Virus vaccine in Addis Ababa, Ethiopia 2023.

**Methods:**

A qualitative research using focused group discussions were used to explore middle adolescent school girls’ perceptions of Human papillomavirus in Addis Ababa Ethiopia from March 2023-April 2023. A focus group discussion guide was employed with potential probes to achieve study objectives. A convenience sampling technique was performed to select study participants. The collected data were transcribed and translated into English and thematic analysis was done by using Atlas-Ti software version 7.5.16.

**Result:**

Two dominant themes emerged from the study; perceived benefits and potential barriers to Human papillomavirus vaccine uptake. Poor awareness, lack of family support, perception of poor vaccine quality, fear of side effects, misconceptions, and myths are potential barriers to the Human papillomavirus vaccine. Some students perceive the vaccine as important in the prevention of cervical cancer, others are not sure about the importance of the vaccine and many students think that the vaccine can cause infertility and other beliefs it can cause diseases.

**Conclusion:**

The perceived benefits of the Human papillomavirus vaccine was; it prevents cervical cancer enhance acceptability and can be an important motivation for Human papillomavirus vaccination among students. The main barriers to vaccine uptake and acceptance were; lack of awareness, misconceptions, and myths, lack of credible information sources of vaccines, perception of poor vaccine quality, Poor family support, and cultural and religious perspectives. We recommend the development of strong collaborations that work on identified challenges.

## Background

Many nations have chosen Human papilloma virus (HPV) vaccination as a primary option for lowering the burden of cervical cancer as a result of regulatory bodies in North America and Europe approving HPV vaccines and the World Health Organization prequalifying them [1]. The HPV vaccination is a preventive vaccine that helps to prevent HPV infections, according to the World Health Organization’s advice [2]. Women’s acceptance of vaccines were reportedly influenced by a variety of factors, including information about the vaccine and attitudes towards the vaccine [3]. Despite the vaccination was the golden method for cervical cancer prevention, most of the adolescents have poor perception of HPV vaccine [4].

The HPV vaccine considerably reduces the risk of infection and the emergence of precancerous lesions as well as the occurrence of cervical cancer. Cervical smear (Pap test) and HPV testing are used to screen for HPV-related lesions all over the world [5]. HPV has been linked to high incidence of cervical cancer. HPV immunization was the best option for protecting women’s from cervical cancer-related illnesses and deaths, especially in resource-limited settings where alternative prevention approaches such as cytology screening or deoxyribonucleic acid (DNA) tests are either difficult to access or are not economically feasible [6]. HPV mostly 16/18, mixed infections and bilharzial infestation, baseline viral load, HR-HPV outcome are the most common risky of recurrence of high-grade cervical lesions [7, 8].

According to a World Health Organization report, two HPV vaccinations are now licensed and marketed. In Ethiopia, approximately 7,000 new instances of cervical cancer are detected each year. It is the second leading cause of cancer death in women aged 15 to 44 years old [9]. Human papilloma virus vaccine uptake among school girls in Ethiopia still was low (44.4%), uptake was majorly related to Hearing about HPV vaccine, availability of awareness creation, and favorable attitude key identified factors associated with vaccination uptake [10].

Despite the fact that Ethiopia is conducting a pilot for HPV vaccine implementation at two sites in Oromia Region, Jimma zone, Gomma woreda, and Tigray Region Ahiferom Woreda with the support of the Global Alliance for Vaccines and Immunization [GAVI], there is no data to show a finding regarding the current status of receivers’ attitude, acceptance, and, in particular, girls’ perception for HPV vaccine. The research aims to improve understanding about HPV vaccination, which could ultimately lead to better health outcomes for young girls. For participants, this research could provide insight into their own thoughts and feelings about the vaccine.

## Methods

### Study area and period

The research was carried out at Lem, Tikur Anbessa, and Raguel secondary and preparatory schools for girls in Addis Ababa, Ethiopia. According to the 2023 reports, Addis Ababa has 11 sub-cities and a total population of 5,460,591 people. Addis Ababa’s current metro area population in 2022 is 4,592,000 a 4.43% growth from 2021. Because the city is the center of the country in many socioeconomic aspects of people’s lives, and because people expect better health services in Addis Ababa than in other regional centers, health facilities in Addis Ababa serve a significant number of people in the city’s surrounding areas and other regional states. Secondary schooling lasts from Grade 9 to Grade 12. The official middle adolescent girl’s school age is from 14 to 17 years old. There are 75 (Government) and 145 (non-government) secondary schools. Among this distribution of female school age population 14–17 years are about 95,064 and Majority of gross enrollment grade 9 to 12 students are female excess for 106, 372 [11].

### Study design

A phenomenological study design was used.

### The participants

#### Inclusion criteria

Girls within age range of middle adolescent [14–17] years, lives in Addis Ababa city, attending secondary school [9–12] grades and had received at least one dose of the HPV vaccination were included in study.

#### Exclusion criteria

Those students who were unwilling to participate, cognitive impairment, other circumstances like anxiety, hearing problem and reduced functional activity were excluded from study.

### Sample Size and Sampling Procedure

The data were collected from six focus group discussion (FGDs), each member containing 8–12 members of female students between the ages of 14–17 years. And the students are grouped in to six equal and homogeneous groups which is based on their age and educational level. The sample size was decided by the saturation point, which implies that enough data was collected for a thorough study. A convenience sampling was used to contact potential participants to inform them of their eligibility to partake. To identify and recruit eligible students we supported from the school leaders and teachers.

This technique assists a small group of participants in learning more about their profound experiences. It enables the thorough explanation of every facet of the phenomenon being studied. In spite of the cases’ variability, it also enables the capture of significant shared or latent patterns [12]. The quality and amount of information in a qualitative research study are referred to as data saturation. It is the moment at which no new information is discovered in the data. It is simply defined as data satisfaction; further data collection is unnecessary. Students were conveniently selected from 9 to 12 classroom.

#### Phenomena of interest

Exploring perception of girls about HPV vaccine.

### Operational definition

#### Middle adolescent secondary school girls

according to world health organization, girls who are enrolled in secondary school, typically age between 14 and 17 [13].

#### Perception about HPV vaccine

the belief adolescents have about the vaccine. Perception of Human Papilloma Virus (HPV) vaccine in this study is defined as the range of beliefs, attitudes and opinions that people have towards the vaccine. This includes knowledge about the vaccine’s safety and efficacy, as well as opinions on the vaccine’s necessity [14].

#### Perceived benefits of HPV vaccine

the individual’s belief that the HPV vaccine is effective in preventing cervical cancer [15].

#### Perceived barriers to HPV vaccine

is the individuals feeling about the obstacles towards acceptance and use of HPV vaccine [16].

#### Awareness to HPV vaccine

In this study as the extent to which members of a target population are aware of the HPV vaccine and its potential benefits, risks and availability [17].

#### Misconception to HPV vaccine

Opinion that is incorrect, misinterpretation or miss understanding of HPV vaccine and its benefits [18].

### Data collection procedures and quality assurance

The data collection tools were prepared by adapting from different literatures. The guideline was written in English, then translated into Amharic by language experts, and finally again translated into English to ensure uniformity. The data were collected from six focus group discussion (FGDs), each member containing 8–12 members of female students between the ages of 14–17 years. And the students are grouped in to six equal and homogeneous groups which is based on their age and educational level.

Focused group discussion was conducted among selected adolescent girl students from secondary schools. The study participants were selected by conveniently sampling method. Female students those who are participated in school Mini media and different school clubs, class representative was selected for the FGD. Those students were considered as they were provided best information.

To identify and recruit eligible students we were supported from the school principal director and teachers. This technique assists a small group of participants in learning more about their profound experiences. It enables the thorough explanation of every facet of the phenomenon being studied. In spite of the cases’ variability, it also enables the capture of significant shared or latent patterns [12]. The quality and amount of information in a qualitative research study are referred to as data saturation. It is the moment at which no new information is discovered in the data. It is simply defined as data satisfaction; further data collection is unnecessary. Students were conveniently selected from grade 9–12.

### Data trustworthiness

It denotes the employment of rigor strategies throughout the investigation. Unlike evaluative norms, which inform the study and offer criteria for evaluating its overall quality, methods for rigor are specific approaches or actions conducted before and throughout the course of the investigation. This includes:

- **Credibility/ Internal Validity**

To maintain credibility, the researcher was actively participating in data collecting. Prolonged interaction, field notes, and observation of participant responses was carried out. Through the interviews, the researcher was acquiring a better grasp of the issue as well as specific parts of the participants’ perceptions.

- **Dependability/ Reliability**

In this study, dependability was attained by describing the research findings, interpretations, and recommendations utilizing an auditable trail to confirm that the inquiry was data-supported and internally coherent. To increase the trustworthiness of all interviews, a voice recorder was employed.

- **Confirmability/ Objectivity**

The employment of an independent coder who went through the transcriptions with the researcher and established a consensus on the themes discovered provided conformability in this study. This was represented in the participants’ voices rather than the researcher’s perceptions. This was supported by the incorporation of an audit procedure in which the researcher describes all research procedures, explains and justifies what they aim to undertake, and makes presentations on the grounds for making judgments.

- **Transferability/ Generalizability**

To guarantee that the study is applicable to other circumstances, the researcher was provide a detailed description of the research approach findings as well as full quotes from individual interviewees. The researcher was request that someone with research knowledge read and choose transcripts at random to identify significant categories so that readers may get a clear picture of the findings.

### Data analysis procedure

Following each session, the transcript was written in Amharic and translated into English. All transcriptions were verified with the audio files and field notes to ensure that everything was translated as accurately as possible. If anything was unclear, the interpreter was listened to the audio recordings again and make the necessary corrections. Atlas-Ti software version 7.5.16 was used to code the English-translated dialogue. Themes were organized based on their concept into categories in the following stage; in this part, the primary codes were classified based on differences and similarities in abstract categories and key concept; and finally, lead to explanation detail description of “Perception of HPV vaccine among Girls” as perceived by the girls. Key concepts classified into two main themes; perceived of barrier and benefits. The continuous analyses of data start from the beginning of codification, and continue until the end of data collection.

## Result

### Participant characteristics

Participants were selected from three schools, which were chosen to be representative of the 220 schools in Addis Abeba. The schools were chosen from a formal list provided by the head department of education and were categorized according to the funding source for their educational institution (public or private); of the three schools selected to participate, one was private and two were public. Students were selected from randomly selected 3 sub cities; Addis Ketema, Bole and Lideta. The schools are Raguel, Tikur Anbessa and Lem secondary and preparatory schools. A total of 6 FGDs were performed with 58 female students, four FGD were from public and two FGD were from private school. Participant’s age ranged from 14 to 17 years. The majority 35(60.3-%) of students were followers of orthodox Christian religion. Most 46 (79%) students are from government schools. One third 42(72%) of students are from grade 11–12.

### Themes and sub-themes (categories) identified

Two major themes emerged from the participants’ narratives about the HPV vaccine. Common patterns embracing were benefits of HPV Vaccine and barriers regarding HPV vaccine among secondary school girls were discussed in all the FGDs. One sub theme emerged from benefits regarding HPV vaccine and six sub-themes were identified within the theme of perceived barriers. It can prevent cervical cancer was perceived benefits for vaccine acceptance and poor awareness, misconception, lack of family support, shortage of credible information, fear of side effect, culture and religion beliefs were perceived barriers.

### Perception of benefits

#### Prevents cervical cancer

There is mixed perception among students about the benefits of HPV vaccine. Some of the students had a favorable attitude and a positive perception about taking the HPV vaccine because they believe it prevents cervical cancer, which is a deadly disease. Few students also reported that their parents encouraged them to take the vaccine regardless of mistaken belief that it would affect uterus. Some schoolgirls thought the immunization prevented cervical cancer, but others connected it to their future reproductive health, placing emphasis on their capacity to procreate.

*A 14-year-old school girl said “We heard that HPV vaccine prevents from cervical cancer. Because of this we want to take the vaccine we don’t want to suffer from this severe disease with no cure” (F4P6).*

*A14- year girl reported that “Some students including me perceive this vaccine has benefit. It can prevent cervical cancer in half of the cases from all who took the vaccine. I mean out of 100-person who took the vaccine 50% of them will be protected. Even though, the protection is not 100%. It is advantageous to prevent cervical cancer” A 16-year school gurl reported (F1P3).*

*A 17-year-old girl reported that “We are potential mothers; this vaccination can reduce maternal death from cancer” (F4P8).*

*“Even though we students don’t know much about the cause and severity of cervical cancer or the vaccine, school girls believe it protects mothers from the disease” A 14-year girl said (F5P1).*

On the other hand, the majority of students think it is not beneficial to take the vaccine.

*A 16-year girl said “Most students think as far as they are not sexually active there is no need of taking the HPV vaccine as it is a sexually transmitted disease” F3P6.*

*16-year girl reported “The vaccine is only useful for people who are sexually active and have multiple sexual partners (F2P3).“*

*“I have no information about with the disease and the vaccine, I don’t think so vaccine is mandatory to receive) A 14-year girl mentioned (F1P4).”*

*A 17-year-old girl “My mother is working in the hospital. I asked her about the benefits of the vaccine. She told me that the HPV vaccine can prevent cervical cancer” (F2P3)*.

### Barriers to HPV vaccination

#### Awareness related barriers

Under this sub theme, students’ knowledge and awareness about HPV vaccine was explored. In the discussion it was mentioned that there were various levels of awareness regarding the HPV vaccine. The Majority of FGDs participants emphasized that there is huge awareness gap regarding the vaccine. It was noted that even though cervical cancer is a major public health problem, the level of awareness of students about the vaccine and disease was not sufficient which can be the main barrier for not taking the vaccine.

Some students have good knowledge about the vaccine when it should be given. Some say the vaccine should be taken before starting sexual contact and the appropriate age to start the vaccination is from age 13–15. Some of them had a good knowledge that multiple sexual contacts were risky for the transmission of HPV.

Most students associate age of vaccination with age of menstrual cycle. They perceive that the disease occurs at age of menses that is why we have to be vaccinated at age 14. Some of the students associate it with reproductive hormone, time to acquire cervical cancer is at age of menses that is raised by most of participants.

*A 17-year girl reported that “………. we didn’t have enough awareness about how the virus can be transmitted and what it causes in our body and how we can prevent the diseases it is impossible to talk about it(F6P4).*

*A 14-year-old told that “We took the vaccine but they didn’t give any awareness about the vaccine. They told as only appropriate age to took vaccine was 13” (F1P6*).

*A 16-year-old 10th class student mentioned that “My perception to the vaccine is poor because during that time of vaccination the health professional didn’t tell us about the importance and side effect of the vaccine” (F6P1).*

*“Some students are not sure that they have enough knowledge about the benefit of the vaccine” 11th class 17th year student reported (F1P2)*.

*A 15-year student mentioned “We don’t have enough knowledge about the disease and the vaccine. Therefore, we are not sure whether the vaccine is important or not.”*

*A 16-year girl said “The major way of disease transmission is multiple sexual partners, if we stop sex with different partner, we can prevent the disease There is no need to take the vaccine”. A 14-year-old reported (F2P3).*

*One of 11th class student mentioned that “I have a fear to be vaccinated; as there are rumors and perceptions that vaccine may kill us” (F2P4).*

*A 15-year-old said “Since this disease is transmitted through sexual intercourse, rather than taking the vaccine it is better to abstain from sex, being faithful, and use condoms” (F3P4).*

*A 10th class student reported that “I have a good knowledge and attitude towards the vaccine”. (F2P7).*

*“Currently, it is one of the diseases that causes death for women worldwide, and if we do not go to the health center early, it causes death, and it has a two-phase vaccine that is administered every six months” A 12th class student reported (F6P3).*

### Misconceptions and myths

The findings of this study revealed a number of persistent patterns of myths about HPV vaccination. The majority of HPV vaccine misunderstandings and concerns were possible impediments to its long-term acceptability. They had the potential to enhance HPV vaccine reluctance (delay in accepting or refusing immunization despite the availability of vaccination services). Some perceives providers inject disease not vaccine. Some of them perceives it can kill them. Most perceives way of transmission through sanitation like exchange of cloths and others reported genetic. Students feared that the vaccine will result in infertility. They associate it with secret population control.

They feel Ethiopia has fast growing population and vaccine is the way given to control it. Due to wide spread of rumors distributed in the community, they perceive the vaccine is illuminate. Participants from group discussed said it can cause blood cancer, due to that she doesn’t want to be vaccinated. Community perceives that it can contradicts with their culture were raised from participants. Most of discussants agreed that it is good to announce the burden of cervical cancer at national level. If community can know burden from disease, they will be aware and start to vaccinate their ladies. They don’t think disease burden is not such much. Participants also Wrongly concept about the transmission and prevention *of* HPV virus.

*A 14-year-old girl reported that “I think it could be transmitted by poor personal hygiene. Mainly not frequent changing of inner dressing (pant)” (F3P1).*

*A twelve-class student reported that “The virus can be transmitted by exchange of cloths and poor sanitation knowing this as a transmissions method we can prevention it. Most women release hormone at age of 14 year; at this age the probability to get cervical cancer is very high I think 14 is right age to start vaccination” (F2P7).*

*A 14-year-old girl said “I think most of us have awareness problem. Some of the students took vaccine to prevent them from disease but some told me they don’t take the vaccine because they are injecting the disease. My mother also not happy on vaccination that is why I am not vaccinated (F2P1)”.*

*“I don’t know it; it is not communicable disease; I think it is transmitted through genetic “an 11th class student reported(F4P5)*.

### Fear of side effects

The majority of students were concerned about the vaccine’s adverse effects. The majority of pupils were concerned about the vaccine’s adverse effects. The vaccination has a major side effect; it may not be a vaccine, but rather a sickness; as a result, students and their families do not want the vaccine, which is stated as…….

*A 12-class student reported that “It could lead to infertility and the vaccine itself is a disease”. “My mom didn’t allow me to be vaccinated some of our families do not allow us to be vaccinated. They think the vaccine has a serious and long-term side effects” (F5P2).*

*“I think the vaccine may make me infertile and afraid of other programmed disease may be injected with or in behalf of this vaccine” (F5P4).*

*A 17-year-old girl “Population of Ethiopia is growing very fast; they are vaccinating us to reduce Ethiopian population because vaccine brings infertility” (F4P5).*

*A 14-year girl mentioned “Most of my friends say vaccination is not important we haven’t seen any one at victim of the disease, but that is not the reason because generally the problem is whole the country and glove so we have to take vaccine” (F1P4).*

*A 15-year-old said “I know nothing about the disease. I only heard about the vaccine in the school and on media. When the vaccinators come to vaccinate, I refuse to take because I was misinformed about the vaccine from my friends and my family. Even I didn’t get permission to be vaccinated. There is rumor in school students about the bad sides of the vaccine. Most of the time these rumors are personal predictions of the negative effect of the vaccine. My families also have such perception” (F3P4).*

*A 14-year-old girl “We are hearing medical and procedural errors. Like that I’m afraid of unnecessary medication or vaccine injection” (F5P6).*

*A17-year old girl “Fear of health effect of the vaccine. I afraid of the vaccine may have serious side effects. Because of this I didn’t take vaccine” (F5P7).*

*A16-year old girl said “When the vaccine came to our school, the vaccinators told us to inform to our families to get permission. Then I told my families. Fortunately, my mother knows about the vaccine, I didn’t face any challenge. She allows me to be vaccinated. However, some of my friends’ families didn’t give permission to be vaccinated. The reason behind were, thinking that the vaccine could results infertility and cancer like leukemia” (F5P3).*

*One of 11th class student mentioned that “I don’t know it’s said effects that is why I am not vaccinated” (F2P4).*

*A twelve-class adolescent girl said that “For feature they should teach benefits and side effect of the vaccine unless it should be stopped. I don’t know how this vaccine works that’s why I am not vaccinated. There were rumors that drug is evil act so detail awareness should be given to enhance confidence on vaccine” (F1P5)*.

*A 12-class student reported that “I fear the side effect of the vaccine and afraid of other programmed disease may be injected with or in behalf of the vaccine. Because of this t I was not vaccinated” (F5P4).*

### Perception of poor vaccine quality

Participants perceive that vaccine given to poor country has poor quality that can affect their health and they don’t want to be injected.

*“Some think that the vaccine we get relates with our economic status. They think the quality of the vaccine may be under standard or suboptimal quality. Since our economic status is low and the cost of the vaccine is high, we may get the vaccine quality according to our economic standard. Cheap cost vs low quality of the vaccine” A 12-class student reported that (F5P4).*

*A 15-year-old girl said that “The community lacks awareness about the benefits of the vaccine. The community perceives the vaccine negatively” (F5P5).*

*A 16-year student mentioned “Some rumors can make us fear of the vaccine because they talk is illuminate not vaccine for prevention. We don’t know it; I think it is not good “(F4P4).*

### Shortage of credible information

HPV believed that credible information sources were necessary due to the large volume of conflicting information available in the community, which can be very confusing and may have a questionable evidence-base. Furthermore, many ‘myths and ‘misconceptions or out-of-date information continue to circulate. Participants stated there is a lack of coverage about HPV vaccine in the local media. In addition, the social media was good way for awareness creation in young population.

Most participants reported that media and government should work hard return the burden of the disease. Even school enforces us to be vaccinated rather giving credible and trustful information regarding vaccine, student asked why they are vaccinated they repeatedly they are vaccinated due to school enforced them to be vaccinated. Vaccine providers enforce us. Most students recommended that school should give timely and trustful information source in addition to health professionals.

*A17-year 12th class student mentioned “I think there is information dissemination problem. For example, detail information is not given. The health personnel themselves do not have confidence about the vaccine. Even they don’t accept the value of the vaccine. The other is there are rumors that needle injury may happen. I myself refused to take during my first exposure of vaccine fearing needle injury. In most of our mind there is a negative idea. I don’t think that anyone who took the vaccine know the benefits of the HPV vaccination. There are information’s on TV and the like Medias but they are warning information. I heard from one TV channel that women should be vaccinated otherwise the disease is very horrible. So, women going to be vaccinated fearing the disease without having the knowledge about the benefits of the vaccine” (F3P9).*

*A 15-year-old said “Lack of information; For instance, I didn’t take the vaccine because I didn’t have information about the vaccine and the disease. The way information was disseminated is also has impact. We young people stay most of our time on social media. You disseminate information on TV but we are on fb, TikTok and the likes” (F3P4).*

*“We were vaccinated because someone come to our school and told as if you are not vaccinated you my get cancer, it may further bring financial and health suffering” (F1P1).*

*A14- year girl said that “We were vaccinated due to school enforced us to vaccinate without any information on it” (F1P3).*

*A 15-year-old girl mentioned that “Since our community have great value for their religious aspects, it is better to give information through their religious organization and persons” (F3P6).*

*A 14-year-old said “We get information about HPV only at the time of vaccination, I think they come at every 6 months and they give information before giving vaccine (F4P8).*

*A 9th class student mentioned that “Mass media like television should give detail information about symptoms, causes and other factors related disease. They should also teach about vaccine. if we know more, we will vaccinate without confusion” (F4P3)*.

*A 17-class girl mentioned that “……. most students were not willing to take the vaccine because the health professionals didn’t give enough information about the vaccine, they only tell us which age group can take the vaccine(F6P1).*

*A15-year old girl mentioned that “Government and media should increase awareness creation, because there are bad rumors about vaccine they distribute easily in community” (F2P5).*

### Culture and religion

Students thinks vaccine is culturally unacceptable. They think their community perceives vaccine can bring infertility that is unacceptable in community and hinders them from receiving vaccine. Basically, peoples in community perceive a cultural think vaccine come to Ethiopia were poor and it brings infertility.

*A 16-year-old girl reported that “Students perceive it contradicts to their culture. They also think that the vaccine unacceptable religiously. Our treatment seeking behavior is poor. So, do you think we do care about vaccine? Students negatively perceive the vaccine(F5P9).*

### Lack of family support

Students repeatedly reported that their family as important part of support and guidance for vaccination. Family perception and knowledge about HPV vaccine has effect on the perception of their children. Those who have educated and knowledgeable family has better opportunity to receive vaccine. Some students reported that family also agrees that vaccination can prevent cervical cancer. Students’ belief that they are potential mothers; taking vaccine is one way to reduce maternal death. Few of students discussed with family, families were agreed that it can prevent disease. Most students don’t get HPV vaccine due to family and community poor perception and beliefs.

*A 16-year-old girl reported that “When the vaccine came to our school, the vaccinators told us to inform to our families to get permission. Then I told my families. Fortunately, my mother knows about the vaccine, I didn’t face any challenge. She allows me to be vaccinated. However, some of my friends’ families didn’t give permission to be vaccine. The reason behind were, thinking that the vaccine could result in infertility and secondary cancer like leukemia” (F5P9).*

*A16-year old girl said “We didn’t discuss about because I and my families do not have any information about the HPV and the vaccine. We discuss another health-related issue and my family help in health-related issues but we didn’t talk about HPV” (F5P3).*

*A 17-year-old girl said “We don’t have awareness to be vaccinated. For instance, I’m not vaccinated because I haven’t knowledge about the benefit of the vaccine. And many of our community perceive as it ends up with infertility; because of this my mom told me no to vaccinated” (F5P2).*

*A 17-class girl mentioned that “………only me and my family were watching TV and the program was about HPV and we discussed together. they give me advice about the importance of the vaccine and said there is no side effect” (F6P1).*

*A16-year old girl mentioned that “My mother prohibited me taking the vaccine because she hears from the community that the vaccine could induce infertility another disease” (F5P3).*

## Discussions

This qualitative study investigated among middle adolescent school girls’ perceptions of Human Papilloma Virus immunization in Addis Abeba, Ethiopia. The perceived benefits and potential barrier to HPV vaccination were explained major themes from the study. Insufficient knowledge, a lack of family support, a belief that the vaccines have poor quality, fear of side effects, misconceptions and myths were all mentioned in this study as potential barriers to HPV vaccination.

The result of current study explained that HPV vaccination was understood beneficial to protect them from severe incurable disease as potential motivator for vaccination. Students believed that the vaccine served as a disease preventative and as a safeguard for the girls’ future reproductive health. This is consistent with many findings from earlier investigations [19–21]. It also reported that students perceived benefits like prevent cervical cancer and prevention of disease was the best motivators to enhance desire to be vaccinated which is congruent with previous finding in different settings [21–23].

The HPV vaccine considerably reduces the risk of infection and the emergence of precancerous lesions as well as the occurrence of cervical cancer. HPV Vaccination were viewed as protection against deadly diseases, which was one of the main reasons for girls’ and parents’ acceptance of HPV vaccination in the study which is similar with previous study [5, 24]. Participants in all FGDs emphasized that there was a significant knowledge gap regarding the HPV vaccine: some believe it can kill, the majority believe that poor hygiene and clothing exchange are the main ways that HPV is spread, and some reported genetics, which is similar to a previous study conducted in the Wolaita zone [25].

In this study, girls’ awareness of HPV was extremely low, which is similar to findings from earlier investigations [26, 27]. In this study, it was often noted that there was a lack of knowledge about the HPV vaccine as well as misconceptions and false information. Contrary to a study conducted in Lagos and Nigeria, where students were shown to have good understanding of the HPV vaccine [20].

It was frequently mentioned in this study that there was a lack of understanding of the HPV vaccine as well as misconceptions and misleading information; contrast to a study carried out in Lagos and Nigeria that revealed pupils to have a strong grasp of the HPV vaccine [22]. Even though most of students have gaps on HPV transmission some knows multiple sexual partners is cause of transmission which is similar with study done at California State University showed multiple sexual partner were the way of HPV transmission [23].

In this study perception of vaccine can kill them which is congruent to study done in Wolaita zone they perceives it can kill them, perceives way of transmission through sanitation like exchange of cloths and reported genetic [25]. It is also reported that some students are aware that HPV is a common cause of cervical cancer in women, which is consistent with research conducted in Italy and North India [28–30]. In this study misconceptions, knowledge of side effects, lack of family support are identified as barriers of HPV; which is contradictory to study done in Nigeria in which the vaccine availability is the main barrier [18]. In this study poor vaccine quality and fear of side effect reported the common reasons of barrier which is similar to study finding from Greek [31].

According to this study, access to information about HPV is deemed to be inadequate. The main issue with vaccine acceptance and barriers for vaccine coverage, which is similar to a study done in Saudi Arabia, was information about vaccine safety and efficacy, low access to HPV information in social media, the absence of educational seminars in schools, and a government platform in providing information on HPV infection and promoting vaccination. Jeddah [32]. There were reports of fears and misconceptions like the vaccine causes infertility and illuminate which discouraged women’s from taking the vaccine which is in line with study from Uganda [24].

The best way to reduce myths and misconceptions is to raise community awareness through this, which is consistent with findings from the United States, with television advertisements, social media access, and health professionals being the most frequently reported reliable sources of information about the HPV vaccine [33]. A study from Germany found that the internet was the least probable source to have heard of HPV, which is consistent with the current study [34].

Contrast to the current study, research from Nigeria shows that the cost of the vaccine was the biggest perceived barrier to immunization [35]. According to research, cultural and religious beliefs are a barrier to HPV, which is consistent with the findings of this study.

Similar to a prior study carried out in Nigeria, family support was a significant factor in this study’s decision to recommend HPV vaccine. Inadequate family support was a hindrance to the HPV vaccine [18]. Study done in Surakarta; Central Java showed that students supported by family members are most likely to be vaccinated in comparison to un supported child’s [36].

This study has some limitations, only females who were enrolled in school and who had received vaccinations were included in our study. As a result, selection bias may exist, and results may not be applicable to females who do not attend school. Since the data for this study was gathered after vaccination, there was a chance that some students’ memories of their immunization status would be biased.

## Conclusion

The current study set out to find out perception of HPV among young school girls in Addis Ababa. The perceived benefits like HPV vaccine can prevent cervical cancer, enhanced acceptability and important motivation for HPV vaccination among students. Lack of awareness, misconceptions and myths, a lack of reputable vaccination information sources, a sense of low vaccine quality, poor family support, cultural and religious attitudes were the main barrier for vaccine acceptability and uptake.

We recommend that the development of strong collaborations that works on identified challenges and barriers on HPV vaccinations. Our findings point to the necessity for a comprehensive education program that includes mass media material in order to offer appropriate information to change misconceptions and raise awareness about the HPV vaccine. Global and local partners could support rising awareness and attitude toward HPV vaccination in school girls. Through parent-teacher conferences and in-person seminars with subject-matter experts, educational practices should be adapted to the community’s unique information needs.

## Data Availability

All-important data were available with correspondent authors and shared accordingly reasonable requests. Copy of this document was submitted to Addis Ababa university library, since all graduate students are mandatory to submit document to university.

## Abbreviations

CC: Cervical Cancer
DNA: Deoxyribonucleic Acid
GAVI: Global Alliance for Vaccines and Immunization
HPV: Human Papilloma Virus
IARC: International Agency for Research on Cancer
MOH: Ministry of Health
